# Current Strategies for Prevention of Infection After Uterine Artery Embolisation

**DOI:** 10.1007/s00270-022-03158-3

**Published:** 2022-05-16

**Authors:** Matthew Lukies, Warren Clements

**Affiliations:** 1 Department of Radiology, Alfred Health, 55 Commercial Road, Melbourne, VIC 3004 Australia; 2 Department of Surgery, Monash University, Melbourne, VIC Australia; 3 National Trauma Research Institute, Melbourne, VIC Australia

**Keywords:** Uterine artery embolisation, Leiomyoma, Fibroid, Infection

## Abstract

Uterine artery embolisation (UAE) is a safe and effective procedure for symptomatic uterine fibroids with an estimated rate of post-operative intra-uterine infection of 0.9–2.5%. While rates of infection have remained low over the past two decades, there is variation in infection prevention practices. Intra-uterine infection after UAE may occur via access site haematogenous spread or ascension of vaginal flora through the cervical canal. Although the evidence base is immature, risk factors for infection including previous pelvic infection, hydrosalpinx, endocervical incompetence, diabetes, smoking, obesity, respiratory disease, and immunosuppression should be assessed during the pre-operative consultation with the interventional radiologist to tailor a plan for minimising infection, which may include optimisation of any modifiable risk facts and prophylactic antibiotics.

## Introduction

Uterine artery embolisation (UAE) is an established treatment option for symptomatic uterine fibroids in all locations [1–10] and has shown early success in adenomyosis [11, 12]. UAE offers several advantages in comparison to surgical options (myomectomy or hysterectomy), including shorter average length of hospital stay, shorter recovery period, similar rates of symptomatic improvement, retention of the uterus, and no requirement for general anaesthesia [1–9]. However, UAE has slightly higher rates of long-term reintervention compared to hysterectomy [1–9], and a recent randomised controlled trial suggested that myomectomy may provide better symptomatic improvement after 2 years, but likely not at 4 years [13, 14]. While UAE is a safe and low-risk procedure, infective complications do occur, which may require hysterectomy (0.4–0.7%) if not resolved with intravenous (IV) antibiotics alone [15–20].

Infection prevention is a priority in UAE, as with any procedure, but particularly so given the outcome of an infective complication may be hysterectomy, which many patients presenting for UAE specifically wish to avoid. Overall rates of uterine infection post-UAE have remained low over the past two decades, with published rates of post-UAE uterine infection under 2.5%, with a possibly higher rate for submucosal or intracavitary fibroids (3.4%) [1, 5, 6, 9, 13, 16, 17, 20–23]. The suggested threshold rate of endometrial or uterine infection post-UAE in intersocietal quality improvement guidelines was 2% [24]. Promisingly, a recent 2020 study found a rate of major uterine infection post-UAE of 0.9% [20]. Comparatively, the rate of post-operative uterine infection in myomectomy is under 4% [5, 13] and there is likely no significant difference in the rate of requiring antibiotic treatment post-UAE compared to myomectomy [4, 5]. While uterine infection post-myomectomy commonly presents earlier, uterine infection post-UAE usually presents after 30 days or more.

The purpose of this review article is to summarise the established evidence and relevant consensus-based guidelines on infection prevention strategies in UAE, focussing on the procedure performed electively for symptomatic uterine leiomyomata.

## Mechanism of Infection in UAE

The aim of UAE is to cause selective infarction of abnormal fibroid tissue. As the normal myometrium receives adequate collateral arterial supply, myometrial ischaemia is mostly resolved within 48–72 h after UAE, whereas the abnormal fibroid tissue does not receive sufficient collateral supply and undergoes infarction [25]. This infarcted tissue, void of a blood supply, is susceptible to opportunistic infection from two different sources: skin flora via procedural arterial access [26] or, perhaps more likely, ascension of vaginal flora through the cervix [16, 21]. Intra-uterine infection (endometritis) may also be more likely with submucosal or intracavity fibroids if they undergo infarction and expulsion [16–18, 21]. One retrospective study of fibroid expulsion post-UAE found that expulsion occurred at a mean of 14.8 weeks (3 months), and while the fibroids had a mixed flora of gram-negative and gram-positive bacteria (including *Escherichia coli* and *streptococcus* species), most infections could be managed conservatively [16]. This study also excluded patients from UAE if they had a pedunculated intracavitary fibroid larger than 6 cm due to concerns for infection. Given that passage of infarcted fibroid may occur several months after the procedure, infection risk may have a pattern of immediate post-operative and delayed peaks.

## Antibiotic Administration

The use of routine prophylactic pre- and post-operative antibiotic administration continues to vary, and recommendations in consensus guidelines tend to be include regimens adopted from other gynaecological procedures including hysterectomy or scientific first principles.

### Prophylactic Pre-operative Antibiotics

Updated practice guidelines in 2018 on Antibiotic Prophylaxis during Vascular and IR procedures [26] recommended a routine regimen of prophylactic antibiotic administration for UAE (Table 1) with a class II a classification (where benefit likely greatly outweighs risk) [27]. Prophylactic antibiotics were defined as those given within 1 h prior to creation of an incision. Published collaborative guidelines in 2013 by the UK radiological and gynaecological colleges specifically on UAE stated that use of pre/intra-operative antibiotics is reasonable, given the evidence for prophylactic antibiotic therapy in vaginal hysterectomy, caesarean section and colorectal surgery, and suggested several agents (Table 1) [18]. However, these guidelines also acknowledged that data on use of prophylactic antibiotics in UAE are limited and concluded that their use should be at the discretion of local hospital policy. As background, antibiotic prophylaxis pre-operatively with cefazolin IV is generally recommended for hysterectomy [28].

**Table 1:** Summary of general antibiotic regimen guidelines for UAE and hysterectomy by major interventional radiology and gynaecology societies

Societal guideline/publication	Procedure	Pre-operative antibiotic administration (single dose within 1 h)	Post-operative antibiotic administration
RCOG/RCR 2013	UAE	Metronidazole with a cephalosporin, a quinolone such as ciprofloxacin, gentamicin or amoxicillin	–
SIR/CIRSE/CAIR 2018	UAE	1–2 g cefazolin IV, with alternatives of (i) 900 mg clindamycin IV + 1.5 mg/kg gentamicin; (ii) 2 g ampicillin IV; (iii) 1.5–3 g ampicillin/sulbactam IV. Vancomycin recommended in penicillin-allergic patients)	100 mg doxycycline oral twice daily for 7 d (in women with hydrosalpinx)
ACOG 2018	Hysterectomy	2 g cefazolin IV (patients ≤ 120 kg), 3 g cefazolin IV (patients > 120 kg)	–

*SIR* The Society of Interventional Radiology, *CIRSE* the Cardiovascular and Interventional Radiological Society of Europe, *CAIR* the Canadian Association for Interventional Radiology, *ACOG* the American College of Obstetricians and Gynecologists

Several studies of the efficacy of UAE have commented on use and non-use of pre-operative antibiotic prophylaxis in their descriptions of procedure technique [5, 22], and reported similarly low rates of uterine infection as a complication; however, few studies have directly investigated the specific question of whether prophylactic antibiotic administration decreases the rate of infection, which ideally needs a prospective randomised trial to definitively answer.

### Post-operative Antibiotics

A recent retrospective cohort study of 375 patients demonstrated no significant difference in the rate of uterine infection in patients given a routine post-operative course of antibiotics (500 mg of oral ciprofloxacin twice daily for 5 days) compared to those who were not (1.8% versus 1.3%, respectively) [20]. All patients in this cohort study received a single dose of pre-operative 1–2 g (weight based) cefazolin IV administered within 1 h of the procedure, and 18.3% of patients had fibroids located submucosally. The SIR guidelines recommend a 7-day course of 100 mg doxycycline twice daily in women with hydrosalpinx (Table 1); however, a small retrospective review suggested that this may not be necessary [29].

Alternatively, as uterine artery embolisation is performed via percutaneous femoral or radial artery access, it may be best categorised as “class I/clean” procedure (an uninfected operative wound in which no inflammation is encountered and the respiratory, alimentary, genital, or uninfected urinary tract is not entered) [30]. From this perspective, some have argued that prophylactic use of antibiotics pre or post-operatively is not necessary unless there are additional risk factors present (e.g. history of pelvic inflammatory disease, previous pelvic surgery, endocervical incompetence, hydrosalpinx, submucosal fibroid location, or immunosuppression) [31].

## Pre-procedure Assessment for Clinical Features and Risk Factors of Pelvic Infection

The pre-procedure assessment consultation provides the interventional radiologist with a valuable opportunity to clinically assess the patient and is considered essential in modern IR practice [32, 33]. This consultation allows for individualised planning, including assessment of potential infective risk factors, which are summarised in Table 2.

**Table 2:** Risk factors to consider in pre-UAE consultation

Gynaecological history	Active pelvic infection
	Previous pelvic inflammatory disease
	Previous pelvic surgery
	Endocervical incompetence
	Hydrosalpinx
	Submucosal/intracavity fibroid location
General medical history	Obesity
	Diabetes
	Smoking
	Respiratory disease
	Immunocompromise

Active pelvic infection is broadly understood to be a contraindication to UAE [2]. The UK UAE guidelines recommend that patients undergoing uterine artery embolisation are assessed pre-operatively for any clinical features of pelvic infection and that if features of active gynaecological infection are present, UAE should be postponed until infection is treated or excluded [18]. Such clinical features of active pelvic infection include vaginal discharge, dysuria, pelvic pain, fever, and dyspareunia. Common responsible organisms include chlamydia trachomatis and gonorrhoea, and investigation of suspected gynaecological infection should include high vaginal swabs and first pass urine analysis. Some centres perform routine genital tract swabs prior to UAE, but there are no specific studies supporting this practice and the utility is uncertain [18].

Hydrosalpinx has been traditionally considered a risk factor for infective pyosalpinx and pyometritis after UAE, with a published report in 2004 describing this complication after UAE for symptomatic fibroids in a 50-year-old woman with asymptomatic unilateral hydrosalpinx at the time of procedure and a distant past history of pelvic inflammatory disease (PID) [34]. In 2012, however, a retrospective study of 16 women with pre-existing hydrosalpinx who underwent UAE did not demonstrate any infective complications or pyosalpinx post-operatively, and showed decreased fallopian tube dilatation in a minority [29]. All patients in this study were given a single dose of 1 g IV cefazolin prior to the procedure, but no further antibiotics post-operatively.

Another cohort of patients potentially at increased risk of infective complications post-UAE are those with autoimmune diseases, either from the disease itself or the immunomodulating medications used in treatment, given the evidence that patients with of higher risk of infective complications after non-UAE gynaecological surgery and other surgeries, particularly if their erythrocyte sedimentation rate (ESR) is elevated [35, 36]. However, a 2019 case–control study of 8 patients with systemic lupus erythematosus (SLE) (*n* = 4), Behcet disease (*n* = 2), rheumatoid arthritis (*n* = 1), and Churg-Strauss disease (*n* = 1) who underwent successful UAE procedures demonstrated no infective complications post-operatively [37]. All patients had stable disease on immunosuppressive medications at the time of UAE, and there was no description of antibiotics given pre- or post-operatively.

## Intra-uterine Devices

Women presenting for uterine artery embolisation may have an intra-uterine device (IUD) in place, which may be contraceptive and/or as part of treatment for heavy menstrual bleeding. The rate of pelvic infection is highest within the first month after insertion of an IUD, and then likely returns to a baseline similar to that for women without an IUD in situ [38, 39]. The presence of an IUD has been conservatively considered a risk factor for infection post-UAE and the 2013 UK guidelines recommend removal before the procedure, without a specified time interval [18]. However, a retrospective study of twenty women who underwent UAE with an IUD in situ found no infective complications, with the authors suggesting that the consequences of IUD removal including pregnancy and aggravated bleeding should be balanced against the likely small risk of infection associated with the IUD [40]. Given the paucity of long term data specifically quantifying the infection risk after UAE with and without an IUD in situ, there is variation in practice with some operators opting not to routinely remove IUDs before UAE and others advocating for pre-UAE removal. Considering that the highest infection risk is during the first month after insertion of an IUD, it may be best to avoid performing UAE within 1 month since insertion or removal of an IUD.

## Arterial Access Site & Closure and Embolic Agents

UAE is commonly performed via femoral or radial arterial access, with a recent study demonstrating similar technical and clinical outcomes [41]. Theoretically, access site flora may seed to the uterus via haematogenous spread; however, with no substantial data on rates of access site infection and septicaemia for radial versus femoral access in UAE, the decision should be made by the operator based on experience and individual patient conditions. While use of femoral artery closure devices may result in a slightly higher rate of access site infection [42], there is no evidence that this increases the rate of uterine infection.

A large number of embolic agents and sizes have been trialled and assessed, particularly regarding their treatment efficacy and pain profile, including non-spherical and spherical polyvinyl alcohol (PVA) particles, tris-acryl gelatin microspheres (TAGM) and gelatin sponge; however, no significant differences in infection rates have been reported [43–48]. Scales of pain post-operatively, as well as rates of infarction and expulsion have been measured in some studies; however, there is no current consensus about the best embolic agent to use. Of note, a 10% rate of fibroid expulsion was reported in one study using TAGM as the embolic agent [49]. Uterine necrosis is also a reported, albeit exceedingly rare complication of UAE, possibly related to the use of smaller spherical particles [50]. Overall, however, no studies have specifically investigated the rates of uterine infection with different embolic agents and sizes. While one may postulate that myometrial infarction, fibroid expulsion [18], and pain post-procedure may have a relationship with risk of infection, these questions have not been specifically investigated.

## Post-procedure Care

There is no consensus guideline available containing advice for women after uterine artery embolisation about resumption of sexual intercourse and use of tampons or other intravaginal hygiene products, with the UK guidelines stating that any guidance is not evidence-based and will depend on local practice [18].

Patient follow-up and active surveillance is an important component of post-operative care after UAE. Patients who are at risk of infection may experience early symptoms and it is important to ensure that interventional radiologists are available to consult their patients after treatment in the event of unexpected fever, discharge, or access site infection. Early identification of patients may allow for early intravenous antibiotic treatment and thus an opportunity to potentially prevent infection-related hysterectomy. All discharged patients should have relevant written advice on symptoms to look out for, who to contact in an emergency, and a follow-up appointment date.

## Conclusion

There is a relative paucity of high quality prospective data on infection prevention measures in UAE, with many practices currently based on data extrapolated from other gynaecological and endovascular procedures, adopted from consensus-based guidelines, or based on personal experience. Interventional radiologists should consider different approaches to risk stratify patients and tailor infection-control measures. This includes thorough pre-operative risk assessment, consideration of prophylactic intravenous antibiotic use, and post-operative advice and active surveillance on discharge.

## Summary Points

- UAE is an effective and safe procedure with a low overall rate of post-operative uterine infection (< 2.5%), similar to myomectomy
- Pre-operatively consultation should include assessment for active pelvic infection and hydrosalpinx, as well as risk factors including previous pelvic inflammatory disease, endocervical incompetence, diabetes, smoking, obesity, respiratory disease, and immunocompromise
- Uterine infection post-UAE likely occurs via procedural arterial access or ascension of vaginal flora through the cervix
- There may be higher risk of uterine infection after UAE of submucosal or intracavitary fibroids, and infection can occur 1–3 months post-embolisation with expulsion of fibroid material
- Pre-procedure (within 1 h) prophylactic antibiotics are generally recommended (typically IV cefazolin); however, there isn’t a strong evidence base
- Post-procedure antibiotics in the absence of risk factors (e.g. hydrosalpinx) are likely unnecessary
- Removal of an IUD prior to UAE is likely unnecessary; however, it may be best to avoid performing UAE within 1 month since insertion of an IUD
- There is no strong evidence for superiority of radial or femoral access or any specific embolic agent regarding infection post-UAE
- There is no strong evidence that use of artery closure devices affects the risk of uterine infection
- Guidance about post-procedural personal care is not evidence-based and will depend on local practice
